# A Markov chain model for studying suicide dynamics: an illustration of the Rose theorem

**DOI:** 10.1186/1471-2458-14-625

**Published:** 2014-06-19

**Authors:** Paul Siu Fai Yip, Bing Kwan So, Ichiro Kawachi, Yi Zhang

**Affiliations:** 1 Department of Social Work and Social Administration, The University of Hong Kong, Hong Kong, Hong Kong; 2 The Hong Kong Jockey Club Centre for Suicide Research and Prevention, The University of Hong Kong, Hong Kong, Hong Kong; 3 Chern Institute of Mathematics, Nankai University, Tianjin 300071, China; 4 Department of Social and Behavioral Sciences, Harvard School of Public Health, 677 Huntington Ave., 7th floor, Boston, MA 02115, USA

**Keywords:** An illness and death model, Markov chain model, Suicide, Rose theorem

## Abstract

**Background:**

High-risk strategies would only have a modest effect on suicide prevention within a population. It is best to incorporate both high-risk and population-based strategies to prevent suicide. This study aims to compare the effectiveness of suicide prevention between high-risk and population-based strategies.

**Methods:**

A Markov chain illness and death model is proposed to determine suicide dynamic in a population and examine its effectiveness for reducing the number of suicides by modifying certain parameters of the model. Assuming a population with replacement, the suicide risk of the population was estimated by determining the final state of the Markov model.

**Results:**

The model shows that targeting the whole population for suicide prevention is more effective than reducing risk in the high-risk tail of the distribution of psychological distress (i.e. the mentally ill).

**Conclusions:**

The results of this model reinforce the essence of the Rose theorem that lowering the suicidal risk in the population at large may be more effective than reducing the high risk in a small population.

## Background

Suicide has become a major public health issue throughout the world. About a million people kill themselves every year, and more than half of these cases occur in Asia [1,2]. It is estimated that about 2.5% of loss of disability-adjusted life year (DALY) is due to suicide and deliberate self-harm [3]. Suicide is especially a major concern in Asia due to its large population size with a relatively high suicide rate and limited resources in suicide prevention [3,4]. In response to the growing concern, different national strategies have been implemented in a number of countries to reduce the number of suicides and the results are somewhat mixed [5]. The programs in Australia, Finland, Norway and Sweden had little or no impact on reducing suicide rates among youth and the general population. However, some more promising results have recently observed in South Korea and Taiwan. There are continuously debates in identifying cost-effective approaches in preventing suicides. Suicide has traditionally been viewed as a mental health issue that is addressed primarily through clinical intervention, especially by providing services for the treatment of depression and other mental illnesses (bipolar disorder, schizophrenia). However, it has been suggested that the role of mental illness in suicide risk is not as significant as expected, especially in the East [1,6-8]. Approximately two-thirds of all people who commit suicide did not receive any specialist psychiatric care in the year before their death [9]. The World Health Organization (WHO) and many national suicide prevention strategies (for example, those in the USA, Austria, Australia, Ireland, New Zealand, and the UK) have proposed a public health approach for suicide prevention, rather than treating it as a medical problem only [3,10-13]. The public health approach involves three layers of intervention: universal, selective and indicated. This public health approach acknowledges the importance of both high-risk and population-based strategies of suicide prevention, and requires a multi-sector effort to tackle the problem at multiple levels: in the community (universal strategies), among specific population subgroups (selective strategies), and among those at a particularly high risk of suicidal behavior (indicated strategies). The public health approach is particularly apt for suicide prevention in Asia where mental health services have not been well developed and awareness and services for depression and mental illness in the community remains inadequate. The Rose Theorem states that a large number of people exposed to a low risk may generate more cases than a small number of people who are exposed to a high risk [14].

Here we adopt an illness and death model [15-17] to model the transition of suicide risk in the population. We make use of the Hong Kong mental morbidity and suicide data to illustrate the applicability of this model. Some empirical results to demonstrating the effectiveness of suicide prevention effort by modifying some parameters of the Markov model will be provided. The underlying framework of this study is not only applicable to suicide research, but could also be extended for other disease prevention purposes and public health challenges.

## Methods

### A suicide dynamic model

**Figure 1:**
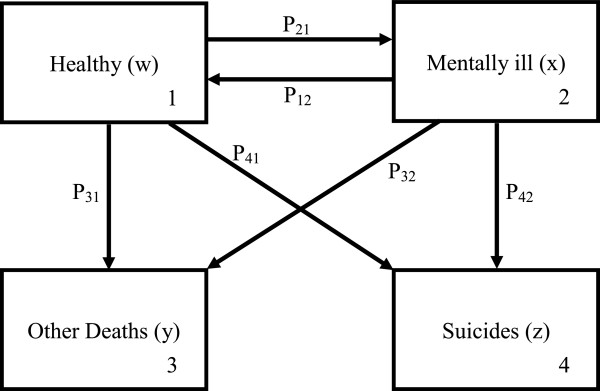
An illness and death model without replacment.

We attempt to model the dynamics of suicide rate, using a Markov chain type model [15,18]. It is assumed that the suicide risk for people with history of mental illness is significantly higher than that of the general population [19]. Our population model consists of four groups: (i) healthy population, (ii) population with mental illness considered to be at high risk, (iii) death due to suicide, and (iv) death due to other causes (not suicide). Every year, we assume a random portion *p*_*21*_ of healthy people would be diagnosed with mental disease; some of whom, *p*_*31*_, would die due to other causes (not suicide), while *p*_*41*_ would die of suicide. Likewise, *p*_*12*_ of patients would recover, and *p*_*32*_ and *p*_*42*_ would die, respectively, from other causes and suicide among the mentally ill. Thus our model can be summarized by Figure 1 and the corresponding evolution equation can be written as:

$$\left(\begin{array}{c} w_{n+1}\\ x_{n+1}\\ y_{n+1}\\ z_{n+1} \end{array}\right)=\left(\begin{array}{c} 1-p_{21}-p_{31}-p_{41}\\ p_{21}\\ p_{31}\\ p_{41} \end{array}\begin{array}{c} p_{12}\\ 1-p_{12}-p_{32}-p_{42}\\ p_{32}\\ p_{42} \end{array}\begin{array}{c} \;0\\ \;0\\ \;1\\ \;0 \end{array}\begin{array}{c} \;0\\ \;0\\ \;0\\ \;1 \end{array}\right)\;\left(\begin{array}{c} w_{n}\\ x_{n}\\ y_{n}\\ z_{n} \end{array}\right),$$

where *w*_*n*_, *x*_*n*_, *y*_*n*_, *z*_*n*_ are respectively the population that is healthy, mentally ill, dead people due to other causes, and dead people due to suicide, in the n-th year.

**Figure 2:**
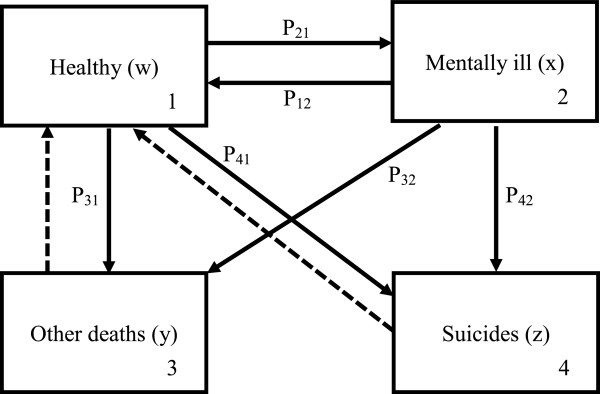
**An illness and death model with replacement**.

It is easy to see the fate of such a dynamic system. As it is a closed system, the dead always remain dead, the total living population (healthy + mentally ill) would decay exponentially. Therefore the dynamics of such model is not particularly interesting.A more interesting model is to assume that the number of deaths removed from the population every year is to be replaced by newborns as depicted in Figure 2 and make sure the dynamic system is always alive.

Hence, with the replacement we can write down the following evolution equation:

(1) $$\left(\begin{array}{c} w_{n+1}\\ x_{n+1}\\ y_{n+1}\\ z_{n+1} \end{array}\right)=\left(\begin{array}{c} 1-p_{21}-p_{31}-p_{41}\\ p_{21}\\ p_{31}\\ p_{41} \end{array}\begin{array}{c} p_{12}\\ 1-p_{12}-p_{32}-p_{42}\\ p_{32}\\ p_{42} \end{array}\begin{array}{c} \;1\\ \;0\\ \;0\\ \;0 \end{array}\begin{array}{c} 1\\ \;0\\ \;0\\ \;0 \end{array}\right)\;\left(\begin{array}{c} w_{n}\\ x_{n}\\ y_{n}\\ z_{n} \end{array}\right).$$

By replacing of the same number of deaths into the healthy stage in order to keep the system alive, it is straightforward to verify that the equation

(2) $$\left(\begin{array}{c} 1-p_{21}-p_{31}-p_{41}\\ p_{21}\\ p_{31}\\ p_{41} \end{array}\begin{array}{c} p_{12}\\ 1-p_{12}-p_{32}-p_{42}\\ p_{32}\\ p_{42} \end{array}\begin{array}{c} \;1\\ \;0\\ 0\\ \;0 \end{array}\begin{array}{c} \;1\\ 0\\ 0\\ \;0 \end{array}\right)\;\left(\begin{array}{c} w\\ x\\ y\\ z \end{array}\right)=\left(\begin{array}{c} w\\ x\\ y\\ z \end{array}\right).$$

which has a unique solution up to a normalization constant, namely:

(3) $$\left(\begin{array}{c} w\\ x\\ y\\ z \end{array}\right)=\frac{Q_{0}}{R}\left(\begin{array}{c} p_{12}+p_{32}+p_{42}\\ p_{21}\\ p_{31}\left(p_{12}+p_{32}+p_{42}\right)+p_{32}p_{21}\\ p_{41}\left(p_{12}+p_{32}+p_{42}\right)+p_{42}p_{21} \end{array}\right).$$

Where *R* = (1 + *p*_31_ + *p*_41_)(*p*_12_ + *p*_32_ + *p*_42_) + *p*_21_(1 + *p*_32_ + *p*_42_), and *Q*_*0*_ is the initial total population size (for example, say around 7 million for Hong Kong in 2011). In other words, Eq. (3) is the unique equilibrium of the dynamic system of people in various stages in the Markov model in Figure 2. Furthermore, given the explicit formulas in Eq. (3), the coefficients of assessing the effects on the number of suicides can also be computed by simply taking partial derivatives.

Here, we are particularly interested in the change in the suicide number. So we take partial derivatives of *z* with respect to parameters, *p*_*ij*_, and evaluate Δ*z* of the following relations. For example, in order to access the change of the number of suicides by modifying a unit of *p*_*41*_, (the proportion of individuals who died from suicide among the healthy ones), we can use the following equation:

(4) $$\Delta z\approx \Delta p_{41}\frac{\partial z}{\partial p_{41}}.$$

And similarly defined for the other parameters, *p*_*ij*_.

## Results

Based on the morbidity data of mental illness supplied by the Hospital Authority of the Hong Kong Government (2012), there were about 150,000 individuals who have received psychiatric service in the government funded hospitals. And there were about 20,000 new psychiatric cases out of the remaining 6.85 million healthy persons annually. Based on the Coroner court files it is estimated that about 30% of the population have received psychiatric treatment and the suicide rate for mentally ill patients was estimated to be 250 per 100,000, while the suicide rate among the non-psychiatric person is about 8.34 per 100,000, i.e., a 30 times differential between psychiatric and non-psychiatric person, which is quite consistent with figures in other countries [20]. The recovery rate from mental illness was estimated at 6.67 per 100 persons and the overall death rate is about 6.0 per 1000 [21]. Hence, the estimated values of the coefficients of *p*_*ij*_ for the Markov model in Figure 2 are given as follows:

*p*_*21*_ = 0.00286 (incidence rate of mentally ill),

*p*_*12*_ = 0.0667 (incidence rate of recovery among the mentally ill),

*p*_*31*_ = 0.006 (death rate from causes other than suicide among the healthy),

*p*_*32*_ = 0.012 (death rate from causes other than suicide among the mentally ill),

*p*_*41*_ = 0.0000834 (suicide rate among the healthy),

*p*_*42*_ = 0.0025 (suicide rate among the mentally ill),

and *Q*_0_ = 7 million in 2012.

From Eq. (3): then one gets

*w* = 0.960 × Q_*0*_ (healthy)

x = 0.0338 × Q_*0*_ (mentally ill)

y = 0.00616 × Q_*0*_ (death from causes other than suicide)

and z = 0.000165 × Q_*0*_ (suicide).

In other words, the Markov dynamic system suggested that the percentage of mentally ill rises from the initial stage of 2.14% and stabilizes at about 3.4% of total population in an equilibrium stage, and the risk of suicides stabilizes at about 16.5 per 100,000 and the death rate from other causes 6.16 per 1,000, which are quite consistent to the recent figures [21].

Suppose that the suicide risk for the healthy population can be decreased via a population-wide mental health promotion program, say, by 1 per 100,000, then according to Eq. (4), the number of suicide deaths decreases by approximately

$$\Delta z=\frac{1}{100000}\times 0.960\times Q_{0}=67.2$$

i.e., a reduction of 67 suicides per year. Correspondingly, if the suicide risk for the mentally ill population can be decreased by the same amount, 1 per 100,000, then the number of suicide deaths decreases by

Δz=2.3

The magnitude of the difference is about 30 times (67.2/2.3) between the non-mentally ill and the mentally ill groups. If the cost of making reduction of risk for health and mentally ill is the same, it would lead to the same reduction in suicide number for producing 30 times effects in the mentally ill population with reference to the non-mentally ill group. This is due to the relatively smaller population size of mentally ill in comparing to the healthy population.

Likewise, the suicide rate for different sets of parameters can be computed. Here, we give the number of suicide (*z*) and its changes (Δ*z*) against variations of the suicide risks of healthy (*p*_*41*_) and mentally ill population (*p*_*42*_), with other parameters held constant as above.

Table 1 shows that there is not much difference in Δ*z* and Δ*p*_*42*_ under the given scenarios. For every 1 per 100,000 change in *p*_*41*_, the reduction of suicide cases (Δ*z*) is about 67 people and the magnitude of the difference between changing the risk in the healthy versus the mentally ill to cause an equivalent reduction in suicide numbers (Δ*p*_*42*_) is about 30 times (this number would be higher when *p*_*42*_ was surprisingly high).

**Table 1:** **Sensitivity analyses of number of suicides respective to the parameters of** ***p***_***41***_**and** ***p***_***42***_

***p***_***41***_	***p***_***42***_	***p***_***42***_**/*p***_***41***_	***z***	**Δ** ***z***^**a**^	**Δ** ***z*/*z***^**a**^	**Δ** ***p***_***42***_^**b**^
						**(×10**^**-5**^**)**
*Reference conditions*						
0.0000834	0.0025	30	1152	67	5.8%	29
*p*_*42*_*fixed at 0.0025*						
0.00001	0.0025	250	659	67	10.2%	29
0.0001	0.0025	25	1264	67	5.3%	29
0.0005	0.0025	5	3950	67	1.7%	29
*p*_*41*_*fixed at 0.0000834*						
0.0000834	0.0001	1	584	67	11.5%	28
0.0000834	0.001	12	801	67	8.4%	28
0.0000834	0.005	60	1710	67	3.9%	31
0.0000834	0.01	120	2734	67	2.5%	35
*p*_*42*_*/p*_*41*_*fixed at 30*						
0.00001	0.0003	30	140	67	47.9%	28
0.0001	0.003	30	1378	67	4.9%	30
0.0005	0.015	30	6460	67	1.0%	39

Note: results for *p*_*42*_/*p*_*41*_, *z*, Δ*z*, Δ*z*/*z*, and Δ*p*_*42*_ are rounded numbers.

^a^If reduce *p*_*41*_ by Δ*p*_*41*_=1×10^-5^.

^b^Reduction in *p*_*42*_ to obtain equivalent Δ*z* under reduction of *p*_*41*_ by Δ*p*_*41*_=1 × 10^-5^.

## Discussion

The analysis of our model suggested that the effect of reducing a relatively small suicidal risk for a large population is more effective than in reducing suicide risk among the mentally ill. The result is consistent with Lewis et al. [22] who have shown that high-risk (indicative) strategies would only have a modest effect on suicide prevention within a population, even if effective interventions could be developed, and that the UK government’s target for suicide reduction was more likely to be achieved using population-wide strategies aimed at lowering risk among the whole population. In contrast to the high-risk approach, a population based approach shall be more radical and with larger influence on the general population. As envisioned by Geoffrey Rose, the population strategy of prevention seeks to shift the entire population’s distribution of “suicide tendency” toward a more favorable direction. This includes promoting the mental health of individuals who are not currently identified as being above a cut-point or a threshold for mental illness or distress.

Indeed our model identifies, theoretically, a reduction of suicide incidence in the healthy population would be more “effective” but in practice, this would only be true if it is at least as practicable to reduce the incidence in the healthy population as compared to the mentally ill. The cost to preventing suicide among mentally ill by intensive clinical care and management would be high but could be more effective in reducing the number of suicide. However, the impact on reduction of number of suicide would still be small as suicide is still a rare event despite the excessive suicidal risk among the mentally ill. It needs to be at least 30 times more effective than in the general population in order to be able to save the same number of suicides. Indeed those who suffered from mentally ill in our community needs better clinical care and treatment but the impact on the number of suicides would still be limited. Furthermore, there are some promising universal programs, for example, restriction of means [23], responsible media reporting [24,25] and some community based programs [26] have demonstrated their effectiveness in reducing suicide numbers in the community and the cost is not that expensive. However, it does rely on participation and co-operation of stakeholders in the community. It is important to develop and evaluate the effectiveness of some population based suicide prevention strategies. Potential effective population approaches for suicide prevention have been so far understudied. It seems that community based support and participation in preventing suicides is not only desirable but also essential [27-29].

**Figure 3:**
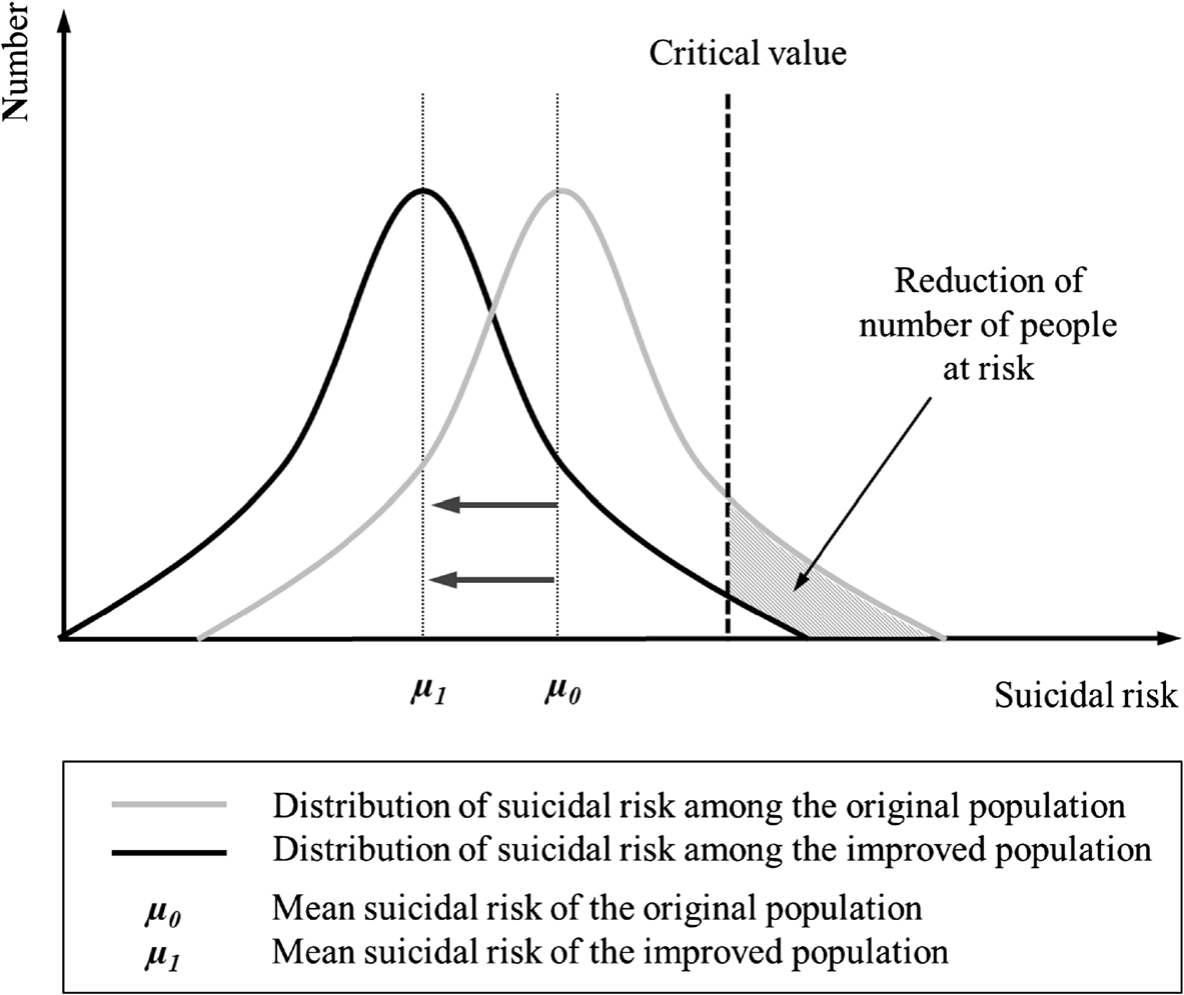
**A schematic representation of the Rose Theorem [**[13]**,**[14]**].**

Furthermore, helping to remove stigma about depression and mental illnesses at a universal level and improving the help-seeking behavior among the needy will also be effective [12]. Sometimes it is difficult to demonstrate the cost-effectiveness of suicide prevention programs in the community. However, the estimated loss of labor productivity due to suicide is high; this is partly due to the increasing rate of suicide among the young and middle aged [12]. Hopefully we shall be able to promote more population based suicide prevention projects which aim at improving wellbeing and help seeking behavior of the community at large. Properly implemented, the Rose theorem illustrates the adage that “an ounce of prevention is better than a pound of cure”. Figure 3 further illustrates this philosophy: by reducing the suicide risk of the population at large (shifting the distribution of the suicidal risk of the whole population to the left), fewer people would experience a high risk of suicide (reduction in the area under the “danger zone”).

There are a few limitations of this study. First, we assume there is a one to one replacement of a death by a living individual. It is a mathematical assumption that allows us to compute the state of equilibrium. It facilitates the computation rather than occurring in real life necessarily. Nevertheless, the essence of the results of the analysis would not be much affected. Second, the results would also depend on the values of *p*_*ij*_, which could be very different in various countries. Hence the results themselves are country specific and may differ between contexts. However, the overall effect should be similar so long as the relationship between the suicide rates among the healthy versus the mentally ill is in the range we have assumed. Also, our calculations do not reflect the differential costing of making a unit change of the death rate from mental ill and healthy individuals. However, it is likely that the cost to reducing a unit of suicide risk among the mentally ill may be more expensive than that of healthy ones but their effect to reducing number of suicides is still uncertain. Our model can be expanded to a formal cost-utility analysis if the specific intervention programs for each strategy (high risk approach versus population approach) could be cost.

## Conclusions

Suicide rate is unlikely to decline sharply through unidimensional effort on preventing the mentally ill patients from suicide, given their high suicidal risk. A more integrated approach of mental health service is needed to be effective to reducing the number of suicide among the mentally ill [28,29]. Furthermore, clinical care for mentally ill is labour intensive and on individual base, its effect on reducing number of suicides has yet to be established. The sensitivity analysis of this model suggests that lowering the suicidal risk in the population at large would be more effective than reducing the high risk in a small population. It underscores the importance of population-based strategies to suicide prevention.

## Competing interests

The authors declare that they have no competing interests.

## Authors’ contributions

PY designed the study. PY, BS and YZ carried out the analysis. PY prepared the first draft of the paper. PY, IK and YZ revised the paper. All authors had agreed with the final version of the manuscript.

## Pre-publication history

The pre-publication history for this paper can be accessed here:

http://www.biomedcentral.com/1471-2458/14/625/prepub
